# Supplementary material for: Dexmedetomidine use for patients in palliative care with intractable pain and delirium: A retrospective study
Source: PLoS One. 2023 Sep 27;18(9):e0292016. doi: 10.1371/journal.pone.0292016 (PMC10530014; doi:10.1371/journal.pone.0292016)
Supplement: Attachment — Submitted filename: Response to Reviewers.docx [file pone.0292016.s001.docx]

**PONE-D-23-07176**

**Dexmedetomidine use for palliative care patients with intractable pain and delirium: A retrospective study**

**PLOS ONE


Please use the space provided to explain your answers to the questions above. You may also include additional comments for the author, including concerns about dual publication, research ethics, or publication ethics. (Please upload your review as an attachment if it exceeds 20,000 characters)**

**Reviewer #1: This is a retrospective review of the use of dexmedetomidine in the treatment of pain and delirium for patients in an inpatient palliative care unit setting. This authors find mixed results in the palliation of those symptoms with some diminution in effect over the course of treatment though accompanied by less utilization of as-needed medication dosing. My overall concerns are as follows:

(1) Terms throughout need operative definitions: "deeply sedating" "proportional rousable sedation" "palliative sedation" need to be defined as objectively as possible to ease comprehensiveness**

This has updated with explanations in the introduction.

**(2)Avoid euphemisms such as "pass away," which also comes from a Christian context and is therefore exclusive of other faith traditions**

We have removed it from the paper.

**(3) Would use person-first language (https://www.cdc.gov/ncbddd/disabilityandhealth/materials/factsheets/fs-communicating-with-people.html) throughout and avoid "palliative care patients" and rather defining them as "patients/people being seen by the palliative care team"**

We have changed this within the paper and in the title.

**(4) Within the methods section, I think much of what you have included in the supplementary materials needs to be included here including more detail about how many pain and delirium assessments were included (all of them or just certain ones during the day); it would also be useful to understand how BP was collected (were all measured BPs collected and averaged? What is the protocol in these units for vital sign collection on the dying? Were there patients who didn't have vital signs collected?), how was delirium assessed, this isn't clear in the manuscript or the supplement; how were pain scores collected for those who can self-report vs. those who could not? This section also needs operative definition for how the authors determined: hypotension (and why was SBP chosen rather than MAP; how was it determined that hypotension was "symptomatic" was this only in those who could verbally report?), reduction in pain (most literature defines a 30% reduction in NPS as clinically meaningful), and reduction in delirium. Were these pre-defined or developed ad hoc?**

Updated the methods section to include exactly how BP was collected. We had looked at number of incidences below our defined SBP that was recorded in the nursing chart and considered each time an episode. We then recorded how many patients experienced any hypotension, mean number of episodes, and mean number of hours prior to first incidence wince the initiation of dexmedetomidine. SBP was chosen because we were using the definition of normal range of 90/60 – 120/80. There was no official pre-printed order at the time of this study so collecting this data was varied based on what was found documented in the nursing charts. Hypotension was determined to be symptomatic in only those patients who verbalized it with more details explained within the paper.

Data collection for delirium was hard to interpret as we found through this study that there were no validated delirium scale being used. We had to base the “scores” from subjective descriptions of the patient’s delirium recorded in their charts and categorized each descriptive symptom as 1 point with a maximum of 5 points total. The decision to do delirium was decided while analyzing the data collected which made the interpretation of dexmedetomidine on delirium difficult.

For pain scores, we only recorded those who can give us their pain score out of 10 and displayed it in our results. We pre-determined a reduction in pain as any reduction from their initial pain score instead of the literature defined 30% reduction.

**(5) Within the methods it would also be helpful to understand what the palliative care teams look like at these sites. Are they multidisciplinary/intraprofessional?**

It is interprofessional and has been updated in the methods.

**(6) Within the results section, it would be helpful to have an idea of what sorts of interventions had been tried and failed before dexamethasone initiation. This would help other teams understand when to consider using it.**

The results have been updated to discuss the other interventions patients were on prior to dexmedetomidine initiation.

**(7) In the results section, I recommend that Tables S1 and S2, be moved into the body of the manuscript rather than the supplement.**

This has been moved to the main manuscript.

**(8) Do these sites have one or more protocols for use of dexamethasone that guide initiation and titration? If so, did they follow them? If so, can they be shared in the supplement for other teams to see and use?**

There was no pre-printed protocol at the time of this study and was only completed after this study was concluded. It is now followed when CSCI dexmedetomidine is started for patients in palliative care. Currently awaiting word from the Clinical Policy Office on whether it can be shared within the supplemental.

**(9) In the results, inclusion of in-line medians and StDev would be useful for things like pain scores, delirium scores, and RASS to help the reader follow along.**

It was felt that comparing the relative pain scores within each patient would be more beneficial than medians due to the small number of patients within the study.

**(10) Within results, it would be helpful to understand if those who were more lightly sedated also required more as needed medications? Was it actually a good thing that they were more awake?**

Around half of the amount of adjunctive medications was the same or reduced in usage when patients were started on dexmedetomidine and it was often requested by the patients wanting to be more alert.

**(11) It would be useful to understand more objectively what was happening with the opioids during this time. Can MEDD/OME be included (and, if so, I will also ask that the conversion factors that are used to calculate MEDD be included in the supplement as well to help with reproducibility)? This is particularly of interest to me for patients 9 & 18 where the discussion section mentions that midazolam was started for pain, which seems concerning if the opioids weren't also being adjusted.**

An updated table with each adjunctive medication’s overall usage when dexmedetomidine was used and compared to 24h after dexmedetomidine was stopped is now in the manuscript. It shows the overall use if it was the same, increased, or reduced when dexmedetomidine was just started to when it has been stopped for 24 hours. In particular for patient 9 and 18, both regularly scheduled methadone was increased in it’s dose.

**(12) Line 143-144 of the discussion introduces new information that the dexmedetomidine was weaned for side effects. Would like to see more about this in the results section. When was that considered? How was it weaned? Is this part of a clinical protocol?**

The discussion section has been updated to include more about when side effects were a cause for weaning.

**(13) I'm not sure that the separate line of best fit figures are necessary. I think the 0-14 day figure would be sufficient if there is more context in the body of the manuscript re: median pain scores.**

We do feel the line of best fit would show each individual patient’s change in pain scores when compared to before they started dexmedetomidine and when they were on it. We did not feel a median score would show the best picture as it’s a small number of study participants.

**(14) If the authors find it relevant, I would be honored to see our case report on dexmedetomidine at EOL also be included as a reference (https://doi.org/10.1080/15360288.2022.2102705).

This is a very important, leading-edge option for palliation and I am excited to see this manuscript. I hope the above recommendations are taken in that spirit of excited anticipation.**

It has now been included as a reference.

**Reviewer #2: This paper presents a case serie for palliative care patients treated with dexmedetomidine (DEX). This use of DEX is promising and at his stage it is good to learn from initial clinical experience. The paper is well written and the discussion reflect the inherent limitations in a retrospective analysis.

Some comments:

1. The authors state that the use of DEX for 8.7 days is longer that in other reports. About at the same time this manuscript was submitted we published a case serie with treatment intervals of median 40 days. Obviously this paper was not available to the authors at the time they prepared their manuscript, but perhaps this information could be added in a revised version. Our paper is available for free at**

[**https://www.ncbi.nlm.nih.gov/pmc/articles/PMC10036681/pdf/40122_2023_Article_485.pdf**](https://www.ncbi.nlm.nih.gov/pmc/articles/PMC10036681/pdf/40122_2023_Article_485.pdf)

We have updated ours to no longer mention longer than in other reports. **2. I miss the development of opioid dose after start DEX; decline, stable or increase? This is needed in order to interpret the potential analgesic benefit from DEX where a part of the success would be to decrease opioids.**

We have updated the manuscript to include a table with individual breakdown of adjunctive medication usage while on dexmedetomidine and 24h post-dexmedetomidine discontinuation.  **3. In the introduction it is correctly argued that DEX may have the patients more arousable than similar sedation with midazolam. However, I miss information about to what extent the patients in this case series after start of DEX was able to have some contact with relatives etc.**

We have updated to include that patients were able to have some contact with relatives.  **4.Hypotension is not defined in the paper. It may be in the supplementary, but let's face it nobody actually read supplementary. The important question is if the hypotension was clinical significant. In our experience it will not be a clinical issue and in this patents setting why really focus on such effects.**

It is correct in that it’s not clinically significant usually but our study found patients who requested the dexmedetomidine to be stopped due to symptomatic side effects. We also included in our study if side effects are seen what the clinical decision making process is to determine if it needs to be discontinued.

**6. page 5 line 95. Small but important typo, not mg but microgram**

Corrected.

**Reviewer #3: Thank you for letting me review this paper on the use of dexmedetomidine in palliative care. The paper is well written and reports on a retrospective chart review of 20 relatively young patients with metastatic cancer disease receiving sc dexmedetomidine due to intractable symptoms in end of life.
The format is more of a short report than an original article. The paper adds to the current scanty literature on the use of dexmedetomidine in palliative care.
My specific comments:
- Abstract, background: Please stress that deep sedation with midazolam in palliative care is limited to end-of-life care.
- Abstract, setting: According to Table S1 all patients had metastatic cancer disease. Adding this information in the text helps to clarify the patient group studied.**

We have added the above 2 points to the abstract.

**- Introduction, third paragraph: I lack some newer references, e.g. the article by Lohre et al in Pain Ther 2023 and the review by Gaertner in Ann Palliat Med 2022 which provides practice recommendations.**

We have updated with the newer references.

**- Methods, third paragraph: I question the detailed description of the data collection instrument and subsequent security routines. Better to replace this with a comprehensive description of the symptom assessment instruments used in the study (mentioned in Introduction and described in supplemental data) together with a short description of statistical methods used (now only described in supplemental data).**

We have updated the methods to include a more detailed description of what was collected.

**- Results, overall: Both Table S1 and S2 provides essential data that is important for the paper. I suggest having them in the paper instead of in supplemental data.**

This has been moved into the manuscript.

**- Results, first paragraph: Adding information that all patients had metastatic cancer and the mean age helps the reader understand the patient group studied.**

This has been added.

**- Results, second paragraph: The authors describe intractable pain as the main reason for starting dexmedetomidine. The paper would benefit from, if possible, having the pain mechanism(s) described for the patients to better understand the different treatment responses observed.**

We did not collect the type of pain mechanisms each patient experienced.

**- Results, third paragraph: It is unclear to me how delirium was assessed and scored, please add this information to Methods. Also, figure 2 is “blurry” and difficult to read. I suggest excluding this figure but keeping the described results in the text.**

Data collection for delirium was hard to interpret as we found through this study that there were no validated delirium scale being used. We had to base the “scores” from subjective descriptions of the patient’s delirium recorded in their charts and categorized each descriptive symptom as 1 point with a maximum of 5 points total. The decision to do delirium was decided while analyzing the data collected which made the interpretation of dexmedetomidine on delirium difficult.

We have removed the figure due to the difficulty in interpreting this data.

**- Results, fourth paragraph: Hypotension and tachycardia were described as the two most common side effects of the treatment. Parallel to this, a gradual decline in PPS scores were seen. Hypotension per se is common in end of life. I lack a discussion around this in Discussion – how could you distinguish treatment related hypotension from the expected hypotension in these dying patients?**

End of life hypotension is commonly seen and with treatment related hypotension it is up to the clinician and often in discussion with the rest of the clinical team to determine if it needs to be stopped such as if the dexmedetomidine was not effective or was running for less than 24h or just had to be dose reduced slightly to see if the side effects stopped. Occasionally if a patient does feel the side effects and requests it to be stopped then that would be another reason to stop it.

**- Discussion, second paragraph, third line: Please correct the sentence.**

Corrected.  **- Discussion, sixth paragraph, fourth line: The authors present data about midazolam CSCI that is not presented in Results. Please add this information to Results, enabling the following discussion.**

Added.

**6. PLOS authors have the option to publish the peer review history of their article (**[**what does this mean?**](https://journals.plos.org/plosone/s/editorial-and-peer-review-process#loc-peer-review-history)**). If published, this will include your full peer review and any attached files.


If you choose “no”, your identity will remain anonymous but your review may still be made public.


Do you want your identity to be public for this peer review? For information about this choice, including consent withdrawal, please see our** [**Privacy Policy**](https://www.plos.org/privacy-policy)**.**

**Reviewer #1: Yes: Kyle P. Edmonds, MD FAAHPM**

**Reviewer #2: Yes: Pål Klepstad**

**Reviewer #3: No**
